# Modelers' Perception of Mathematical Modeling in Epidemiology: A Web-Based Survey

**DOI:** 10.1371/journal.pone.0016531

**Published:** 2011-01-31

**Authors:** Gilles Hejblum, Michel Setbon, Laura Temime, Sophie Lesieur, Alain-Jacques Valleron

**Affiliations:** 1 INSERM, U707, Paris, France; 2 UPMC Univ Paris 06, Faculté de Médecine Pierre et Marie Curie, UMR S 707, Paris, France; 3 AP–HP, Hôpital Saint Antoine, Unité de Santé Publique, Paris, France; 4 CNRS, UMR 6123, Aix-En-Provence, France; 5 Université de la Méditerranée, Laboratoire d'Économie et de Sociologie du Travail, Aix-En-Provence, France; 6 CNAM, Chaire Hygiène & Sécurité, Paris, France; Universita' del Piemonte Orientale, Italy

## Abstract

**Background:**

Mathematical modeling in epidemiology (MME) is being used increasingly. However, there are many uncertainties in terms of definitions, uses and quality features of MME.

**Methodology/Principal Findings:**

To delineate the current status of these models, a 10-item questionnaire on MME was devised. Proposed via an anonymous internet-based survey, the questionnaire was completed by 189 scientists who had published in the domain of MME. A small minority (18%) of respondents claimed to have in mind a concise definition of MME. Some techniques were identified by the researchers as characterizing MME (e.g. Markov models), while others–at the same level of sophistication in terms of mathematics–were not (e.g. Cox regression). The researchers' opinions were also contrasted about the potential applications of MME, perceived as higly relevant for providing insight into complex mechanisms and less relevant for identifying causal factors. The quality criteria were those of good science and were not related to the size and the nature of the public health problems addressed.

**Conclusions/Significance:**

This study shows that perceptions on the nature, uses and quality criteria of MME are contrasted, even among the very community of published authors in this domain. Nevertheless, MME is an emerging discipline in epidemiology and this study underlines that it is associated with specific areas of application and methods. The development of this discipline is likely to deserve a framework providing recommendations and guidance at various steps of the studies, from design to report.

## Introduction

The increased use of mathematical modeling in epidemiology (MME) is widely acknowledged [1]. When data are not there, or not yet there, MME provides rationales in Public Health problems to support decisions in Public Health, and this constitutes one of the reasons for the increased use of MME, For example, some models have been proposed for estimating non observable putative risks of importance in terms of public health, such as the risk of cancer after exposure to diagnostic radiations [2], the residual infectious risks in blood transfusion [3], or the future size of a the epidemic of an emergent disease [4]. MME is also unavoidable in economic studies [5], [6] and has been used for studying the dynamics and control of epidemics of infectious diseases [7]. For example MME has been widely applied to influenza pandemics (see among many references, refs [8], [9], [10], [11]). In addition, MME provides insights on the role of possible determinants of diseases that were overlooked in the traditional epidemiological approaches: for example Christakis et al reanalyzed the iconic Framingham studies and showed that some network sociological properties could explain observed trends in the incidence of several chronic diseases [12], [13].

The above list of key public health domains attests for the diversity and interest of problems for which MME might be involved in supporting public decision. Such an involvement both reassures and worries the decision makers and the public at large who eventually have concerns on the nature of the science of MME. Indeed, the heterogeneity of the methods used in MME, as well as the diversity of the problems addressed raises several questions: is there a simple shared definition for this emerging scientific discipline? Is MME only mainly aimed to answer questions of decision makers, or is it a scientific discipline of its own? What are the criteria of good science in MME? To answer these questions, we chose to collect the opinions of the scientists who actually do MME. We present here the results that were observed on a panel of scientists who participated in a web-based survey investigating the above issues.

## Materials and Methods

### Survey participants

We constituted a panel of researchers who had published academic papers in the field of MME through a search in the Science Citation Index database via the Web of Sciences (Thomson Reuters, NY). In a query made on February 2, 2007, we identified all papers published since 2002 containing the terms “mathematical model*” (* is a wildcard end of the term) in the title, the abstract, or among the keywords and published in a journal belonging to at least one of the six following categories of the Journal of Citation Reports (Thomson Reuters, NY): public, environmental & occupational health; infectious diseases; oncology; medicine, research & experimental; social sciences, mathematical methods; medicine, general & internal. This search retrieved 920 articles. The email address of the corresponding author was documented in the Web of Science records for 529 of the 920 papers, yielding one email address for 482 different authors. We manually searched for the email addresses of the corresponding authors of the 391 remaining articles, which in turn provided a second series of 301 additional email addresses. In the end, 126 of the total 783 addresses returned an error message, being wrong or more likely obsolete. Finally, we obtained a list of 657 authors with a valid email.

We emailed these 657 authors to invite them to participate anonymously in our survey, as we provided each author with an anonymous and personal login. The survey was developed through the Internet using phpESP freeware [14]. Our website hosted the survey and was open during two periods: from October 11 to October 27, and from November 11 to December 8, 2007. During these periods, reminders were sent to all the potential participants.

Each participant was asked to provide his age, background, present academic position and country of work. The survey was completed by 189 participants (29% response rate) who constituted the panel herein analyzed. 49% of the respondents were under 45 years old. Most (75%) participants answered that they had a background in mathematics or physics; 40% were university teachers or researchers, 36% were researchers with institution responsibilities (faculty head, institute head, department head, lab head, research director, or senior researcher), 9% were postdoctoral researchers, and 6% were senior medical doctors. The percentages of participants working in America, Europe, Asia, Africa and Australia, were respectively 46% (including 36% from the USA and 5% from Canada), 38% (including 12% from U.K.), 8%, 2% and 6%.

### Questionnaire

**Figure 1:**
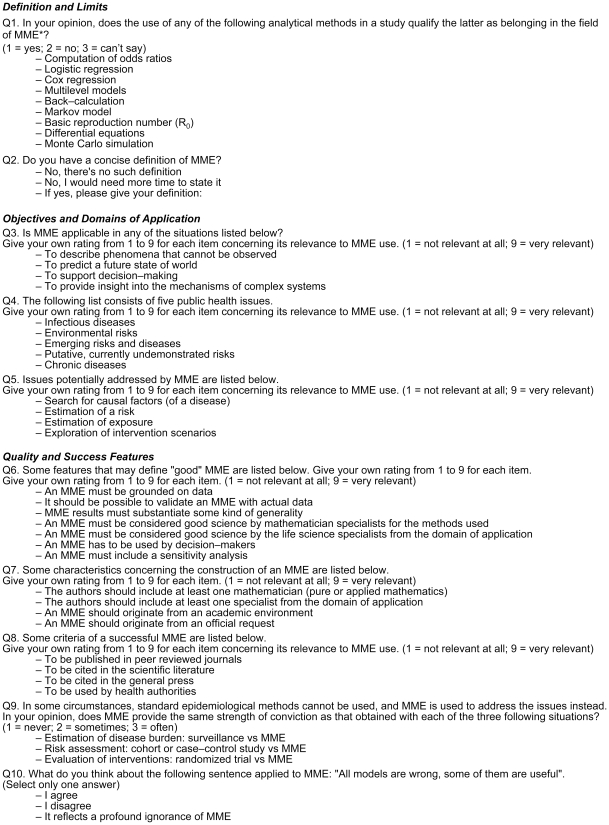
The questionnaire of the survey.

The questionnaire was composed of ten one-minute questions (Figure 1). The answer required for questions Q3 to Q8 was obtained by clicking on a 9-point scale, with 1 corresponding to “not relevant at all”, and 9 to “very relevant”. Questions Q1 and Q2 concerned the author's definitions of a MME. Questions Q3 to Q5 investigated the conceptual objectives of MME (Q3 and Q5) and public health domains of applications (Q4). The five remaining questions (Q6–Q10) explored quality and success features associated with the use of these models: three questions examined criteria for quality (Q6 and Q7) and success (Q8); Q9 evaluated whether the panelists perceived a difference between the strengths of conviction of the results derived from statistical epidemiology and those from MME. Finally, we asked the participants their opinions on the statement “All models are wrong, some may be useful” (Q10), made by the statistician George Box who co-authored the Box and Jenkins method [15].

### Statistical analysis

Answers to Q1 were recoded on an ordinal 3-point scale (“no”, “can't say” and “yes” were respectively recoded as 1, 2 and 3), as were responses to Q9 (“never”, “sometimes” and “often” were respectively recoded as 1, 2 and 3). Considering all the items investigated in Q1 and in Q3–Q9, the respondents' answers to the items of a question (or a group of questions) were handled as measures issued from a complete block design, the first (fixed) factor being the explored item, the second (block, random) factor being the respondent. Therefore, when analyzing the potential differences in the responses to several items, we performed multiple comparisons between all considered items using the Wilcoxon–Nemenyi–McDonald–Thompson procedure recommended by Hollander and Wolfe [16]. This procedure identifies potential clusters of items, with a non-significant (P>0.05) difference between any two items belonging to a given cluster and, conversely, a significant (P≤0.05) difference between any two items belonging to two distinct clusters. R statistical software [17], version 2.9.0, was used for all analyses.

## Results

**Figure 2:**
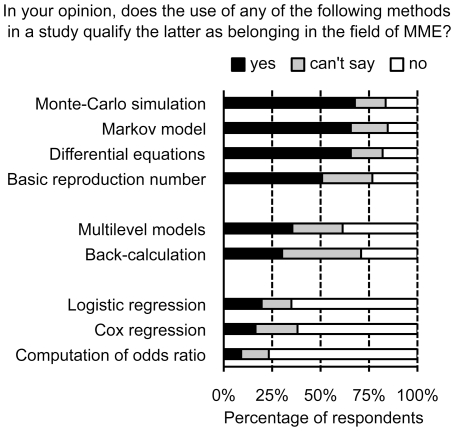
Perceived relevances of nine techniques for qualifying a study in the field of mathematical modeling in epidemiology (MME).

Considering the definition of MME addressed in question Q2, 22% of the respondents answered that no such definition exists, and 60% indicated that they would need (more) time to state it. Question Q1 investigated whether the uses of 9 mathematical or statistical methods frequently used in epidemiology were judged by the panel as characterizing the field of MME. The multiple comparison analysis identified three clusters of items (Figure 2). The first cluster groups items (Monte-Carlo simulation, differential equations, Markov model, basic reproduction number R0) for which most respondents considered that the use of the technique in the analysis qualifies the study as belonging to the MME field. In contrast, standard statistical epidemiological techniques (logistic regression, Cox regression and computation of odds ratio) constituted a second cluster of items for which only a small minority of respondents considered their use is sufficient to qualify the study as belonging to the MME domain. The intermediate cluster was comprised of multilevel models and back-calculation. Multilevel models, which are plain statistical techniques, were placed in this intermediate group, not in the statistical tools' category, and back-calculation was considered by only a minority of the respondents as qualifying a study as belonging to MME.

**Figure 3:**
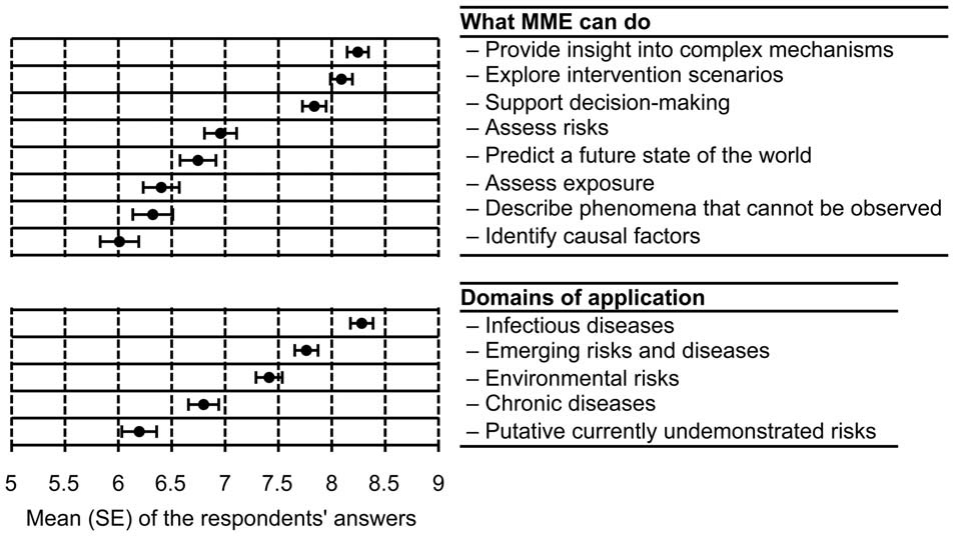
Perceived role of mathematical modeling in epidemiology (MME). Means and standard error (SE) of the responses obtained for each item are shown. Respondents scored each item on a 1–9 scale (1 =  not relevant at all, 9 =  very relevant). Top panel: items from questions Q3 and Q5; bottom panel: items from question Q4. The exact formulations of questions Q3 to Q5 are in Figure 1.

The eight different possible roles and five domains of application of MME suggested were ranked by the participants (Figure 3). The most prominent MME roles identified by the respondents were the capacity to provide insight into a complex mechanism and, in terms of health policy, to explore intervention scenarios and to support decision-making. At the opposite end, the least recognized MME role was the identification of causal factors. The possible use of MME to investigate putative presently undemonstrated risks received a very low rating. Finally, the respondents clearly ranked the different domains of application, putting infectious diseases at the top and chronic diseases at the bottom.

**Figure 4:**
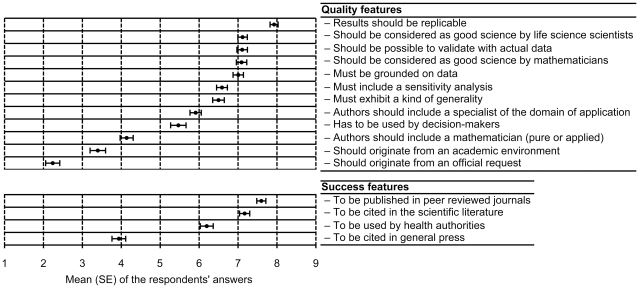
Perceived quality and success features of mathematical modeling in epidemiology. Means and standard error (SE) of the responses obtained for each item are shown. Respondents scored each item on a 1–9 scale (1 =  not relevant at all, 9 =  very relevant). Top panel: items from questions Q6 and Q7; bottom panel: items from question Q8. The exact formulation of questions Q6 to Q8 are in Figure 1.

The last dimension explored in the survey addressed the perceived determinants of a “good MME”, in terms of quality and success (Figure 4). The criteria of the panel are (not surprisingly) academic, as evidenced by the ranking of the quality features. The fact that the MME has originated from an official request got the lowest ranking, and even the fact of being used in practice by decision-makers was rated low. Academic values, again, were considered as the main criteria of success of MME (Figure 4). Indeed, the major perceived success of a model is to be published in good scientific journals, not in the lay press. No significant difference was found between public health domains in terms of the perceived strength of conviction provided by statistical or mathematical epidemiology (Table 1). Box's provocative remark was endorsed by 65% of the respondents, 20% disagreed, and 15% labeled it as “reflecting a deep ignorance of MME”.

**Table 1:** Strengths of conviction obtained by mathematical modeling in epidemiology (MME) versus standard epidemiological methods.

Answer to “Results from MME as convincing as results from standard epidemiological methods?”*	Public health domain		
	Estimation of burden of disease (%†)	Risk assessment (%†)	Evaluation of intervention (%†)
Never	14	17	22
Sometimes	71	68	54
Often	15	15	24

*The exact formulation of the question is in Table 1 (Q9).

† Percent of answers, n = 189.

Multiple comparisons between the 3 public health domains all resulted in non significant differences: estimation of burden of disease versus risk assessment and versus evaluation of intervention, *P* = 0.97 and *P* = 0.81, respectively; risk assessment versus evaluation of intervention, *P* = 0.66.

## Discussion

The response rate to the present survey was 29%, within the range of the rate reported for Web-based surveys (median at 27%) [18]. This rate does not impact our results. The purpose of our work was not to provide figures on the perception of MME in researchers or epidemiologists at large, but to rank these perceptions. For example, the panel researchers' much higher ranking of MME relevance in infectious disease than in chronic diseases can certainly be safely extrapolated. This is the usual situation in epidemiology: absolute values observed on a particular study are difficult to extrapolate, but differences and correlations within the group can be safely extrapolated.

The first question we posed was about the identity of MME, given the large diversity of methods and uses, as underlined by Weinstein et al [19]. The results show that the use of statistical “models”, such as the Cox Model, is not considered by the researchers as qualifying a work as in the field of MME, while conversely, Monte Carlo simulations, Markov models and differential equations are. The use of the basic reproductive number in a paper was also considered as a good indicator of MME, with a more modest score achieved (51%) that might be due to the fact that R_0_ is increasingly viewed as a general epidemiological concept. Conversely, even if back-calculation can be considered as a historical success story of mathematical epidemiology [20], it is however viewed more as a statistical technique, computationally demanding. All in all, mathematical modelers appear to characterize models more by the abstraction of the representation of the epidemiological mechanisms studied (e.g. transmission, immunity) than by the sophistication of the underlying mathematical tools.

The second question aimed at delineating the limits of what models can and cannot do from the point of view of those who produce them. The answers are that MME is more suited for clarifying the laws underlying complex mechanisms, for supporting decision-making, and for exploring intervention scenarios. MME is not perceived as competing with standard epidemiology for the discovery of the causes of the diseases and/or risk factors. Infectious diseases, emerging risks and diseases, and environmental risks, were scored as domains for which MME is highly applicable.

The third question aimed at identifying the determinants of a “good” model. Keeling and Rohani stated that what constitutes a good model depends on the context [21]. Notably, modelers think that decision-making is an important goal of MME but do not consider practical use of their models by health authorities for decision-making as an adequate criterion of MME quality. Modelers acknowledge the gap between their scientific output and public uses of it to which they are not automatically associated. The quality features perceived by the respondents all relate to scientific excellence. The finding that the values of the mathematical modelers in epidemiology are clearly academic has two practical consequences. First, scientific policy makers and proposal evaluators in the field of MME should be aware that these researchers expect that an assessment of their projects, works and even careers be primarily based on their intrinsic scientific quality, not their public health “usefulness”. Second, public health decision-makers should keep that finding in mind, when modelers do not provide quick and simple answers to the problems they pose; above all, modelers are driven in their agendas by the quality and originality of the methods they develop, even when the general direction of their work is oriented by important public health issues.

In conclusion, this study shows that perceptions on the nature, uses and quality criteria of MME are contrasted, even among the very community of published authors in this domain. Nevertheless, MME is an emerging discipline in epidemiology and this study underlines that it is associated with specific areas of application and methods. The development of this discipline is likely to deserve a framework providing recommendations and guidance at various steps of the studies, from design to report. Previous similar proposals in other domains of epidemiology have been successful. For example, CONSORT guidelines in the domain of clinical randomized trials [22], [23], represent a prototype of what could be done in the domain of MME.
